# ﻿Study on species diversity of *Akanthomyces* (Cordycipitaceae, Hypocreales) in the Jinyun Mountains, Chongqing, China

**DOI:** 10.3897/mycokeys.98.106415

**Published:** 2023-07-28

**Authors:** Wan-Hao Chen, Jian-Dong Liang, Xiu-Xiu Ren, Jie-Hong Zhao, Yan-Feng Han

**Affiliations:** 1 Center for Mycomedicine Research, Basic Medical School, Guizhou University of Traditional Chinese Medicine, Guiyang 550025, Guizhou, China Guizhou University of Traditional Chinese Medicine Guiyang China; 2 Institute of Fungus Resources, Department of Ecology, College of Life Sciences, Guizhou University, Guiyang 550025, Guizhou, China Guizhou University Guiyang China

**Keywords:** entomopathogenic fungi, morphology, phylogenetic analysis, Sordariomycetes, spider-pathogenic fungi

## Abstract

*Akanthomyces* species have only been reported from Guizhou and Qinghai Province, with few reports from other regions in China. In this research, the species diversity of *Akanthomyces* in the Jinyun Mountains, Chongqing was investigated. Fourteen infected spider specimens were collected and two new species (*A.bashanensis* and *A.beibeiensis*) and a known species (*A.tiankengensis*) were established and described according to a multi-locus phylogenetic analysis and the morphological characteristics. Our results reveal abundant *Akanthomyces* specimens and three species were found at Jinyun Mountain. Due to its being an important kind of entomopathogenic fungi, further attention needs to be paid to the diversity of other entomopathogenic fungi in Chongqing, China.

## ﻿Introduction

*Akanthomyces* Lebert was established by Lebert for the species, *A.aculeatus* Lebert in 1858. Mains (1950) reported three additional species: *A.ampullifer* (Petch) Mains, *A.angustisporus* Mains and *A.aranearum* (Petch) Mains. Subsequently, several other species have been reported (Samson and Evans 1974; Koval 1977; Vincent et al. 1988; Hywel-Jones 1996; Hsieh et al. 1997; Huang et al. 2000; Mongkolsamrit et al. 2018; Chen et al. 2018, 2019c, 2020b, 2020c, 2022a; Shrestha et al. 2019; Aini et al. 2020; Wang et al. 2023).

Based mainly on phylogenetic analyses, several *Akanthomyces* species (*A.arachnophilus* (Petch) Samson & H.C. Evans, *A.cinereus* Hywel-Jones, *A.koratensis* Hywel-Jones, *A.longisporus* B. Huang et al., *A.novoguineensis* Samson & B.L. Brady, *A.ovalongatus* L.S. Hsieh et al. and *A.websteri* Hywel-Jones) were transferred to the new genus *Hevansia* Luangsa-ard et al. (Kepler et al. 2017). In addition, *Lecancilliumattenuatum* Zare & W. Gams, *L.lecanii* (Zimm.) Zare & W. Gams, *L.longisporum* (Petch) Zare & W. Gams, *L.muscarium* (Petch) Zare & W. Gams, *L.pissodis* Kope & I. Leal and *L.sabanense* Chir.-Salom., S. Restrepo & T.I. Sanjuan were transferred to *Akanthomyces*. As a result, the genus *Akanthomyces* currently consists of 34 species.

*Akanthomyces* is an important genus in entomopathogenic fungi and its diverse bioactive substances have attracted widespread attention (Lee et al. 2005; Madla et al. 2005; Kuephadungphan et al. 2014; Putri et al. 2014; Kinoshita et al. 2017). However, *Akanthomyces* species have only been reported from Guizhou and Qinghai Province and there have been few reports from other regions in China (Chen et al. 2018, 2019c, 2020b, 2020c, 2022a; Wang et al. 2023). In this research, the species diversity of *Akanthomyces* in the Jinyun Mountains, Chongqing was investigated. Several spider-associated specimens were found and a few new *Akanthomyces* strains were isolated and purified. The goal of this research was to identify these new strains by multigene phylogeny as well as by morphological characteristics.

## ﻿Materials and methods

### ﻿Specimen collection and identification

Fourteen infected spider specimens were collected from Jinyun Mountain (29°50'22.14959"N, 106°23'18.0744"E), Beibei District, Chongqing, in May 2021. The surface of each spider body was rinsed with sterile water, followed by surface sterilisation with 75% ethanol for 3–5 s and rinsing 3 times with sterilised water. After drying on sterilised filter paper, the mycelium or a part of the sclerotium was removed from the specimen and inoculated on potato dextrose agar (PDA) and improved potato dextrose agar (PDA, 1% w/v peptone) plates (Chen et al. 2019b). Fungal colonies emerging from the specimens were isolated and cultured at 25 °C for 14 days under 12 h light/12 h dark conditions following protocols described by Zou et al. (2010). The specimens and axenic cultures were deposited at the Institute of Fungus Resources, Guizhou University (formally Herbarium of Guizhou Agricultural College; code, GZAC), Guiyang City, Guizhou, China.

Macroscopic characterisation was determined from PDA cultures incubated at 25 °C for 14 days, including the growth rate of the colony, the presence of octahedral crystals, and the colours of the colony (surface and reverse) were observed. To investigate the microscopic characteristics, a small amount of the colony was removed and mounted in lactophenol cotton blue or 20% lactate acid solution and observed using an optical microscope (OM, DM4 B, Leica, Germany).

### ﻿DNA extraction, polymerase chain reaction amplification and nucleotide sequencing

DNA extraction was carried out using a fungal genomic DNA extraction kit (DP2033, BioTeke Corporation) according to Liang et al. (2011). The extracted DNA was stored at −20 °C. Polymerase chain reaction (PCR) was used to amplify genetic markers using the following primer pairs: ITS4/ITS5 for the internal transcribed spacer (ITS) region (White et al. 1990), LR0R/LR5 for 28s large subunit ribosomal (LSU) (Vilgalys and Hester 1990), fRPB2-7cR/fRPB2-5F for RNA polymerase II second largest subunit (RPB2) (Liu et al. 1999) and 2218R/983F for translation elongation factor 1 alpha (TEF) (Castlebury et al. 2004). The thermal cycle of PCR amplification for these phylogenetic markers was set up following the procedure described by Chen et al. (2021). PCR products were purified and sequenced at Sangon Biotech (Shanghai) Co. The resulting sequences were submitted to GenBank (Table 1).

**Table 1.** List of strains and GenBank accession numbers of sequences used in this study.

Species	Strain	GenBank accession numbers			
		ITS	LSU	RPB2	TEF
*Akanthomycesaculeatus*	HUA 186145	-	MF416520	-	MF416465
*A.aculeatus*	TS772	KC519371	KC519370	-	KC519366
*A.araneicola*	GY29011	MK942430	-	MK955947	MK955950
*A.araneicola*	GY29012	MK942435	-	MK955948	MK955951
*A.araneicola*	GY29013	MK942436	-	MK955949	-
*A.araneogenus*	GZUIFDX2	KU893153	-	MH978185	MH978187
*A.araneogenus*	GZUIFDX1	KU893152	-	MH978184	-
*A.araneogenus*	GZUIFSN1	MH978177	-	MH978186	MH978188
*A.araneosus*	KY11341	ON502826	ON502832	ON525442	ON525443
*A.araneosus*	KY11342	ON502844	ON502837	ON525444	ON525445
*A.attenuatus*	CBS 402.78	AJ292434	AF339565	EF468935	EF468782
***A.bashanensis***	**CQ05621**	**OQ300412**	**OQ300420**	**OQ349684**	**OQ325024**
***A.bashanensis***	**CQ05622**	**OQ300411**	**OQ300421**	**OQ349685**	**OQ325025**
***A.beibeiensis***	**CQ05921**	**OQ300415**	**OQ300424**	**OQ349688**	**OQ325028**
***A.beibeiensis***	**CQ05922**	**OQ300416**	**OQ300427**	**OQ349689**	**OQ325029**
*A.coccidioperitheciatus*	NHJ 6709	JN049865	EU369042	EU369086	EU369025
*A.kanyawimiae*	TBRC 7242	MF140751	MF140718	MF140808	MF140838
*A.kanyawimiae*	TBRC 7243	MF140750	MF140717	MF140807	MF140837
*A.kanyawimiae*	TBRC 7244	MF140752	MF140716	-	MF140836
*A.lecanii*	CBS 101247	JN049836	AF339555	DQ522466	DQ522359
*A.lepidopterorum*	SD05151	MT705971	MT705973	MT727044	-
*A.lepidopterorum*	SD05152	MT705972	MT705974	MT727045	-
*A.neoaraneogenus*	GZU1031Lea	-	KX845703	KX845701	KX845697
*A.neoaraneogenus*	GZU1032Lea	-	KX845704	KX845702	KX845698
*A.neocoleopterorum*	GY11241	MN093296	-	MN097812	MN097813
*A.neocoleopterorum*	GY11242	MN093297	-	MN097814	MN097815
*A.noctuidarum*	BCC36265	MT356072	MT356084	MT477987	MT477978
*A.noctuidarum*	BBH16595	MT356073	MT356085	MT478005	MT477979
*A.noctuidarum*	BCC47498	MT356074	MT356086	MT477988	MT477980
*A.noctuidarum*	BCC28571	MT356075	MT356087	MT478006	MT477981
*A.pissodis*	CBS 118231	-	KM283799	KM283864	KM283822
*A.pyralidarum*	BCC28816	MT356080	MT356091	MT478007	MT477982
*A.pyralidarum*	BCC32191	MT356081	MT356092	MT477989	MT477983
*A.pyralidarum*	BCC40869	MT356082	MT356093	MT477990	MT477984
*A.pyralidarum*	BCC29197	MT356083	MT356094	MT477991	MT508840
*A.sabanensis*	JCh041	-	-	KC875224	KC633274
*A.sulphureus*	TBRC 7247	MF140756	MF140720	MF140811	MF140841
*A.sulphureus*	TBRC 7248	MF140758	MF140722	MF140812	MF140843
*A.sulphureus*	TBRC 7249	MF140757	MF140721	MF140734	MF140842
*A.thailandicus*	TBRC 7245	MF140754	-	MF140809	MF140839
*A.thailandicus*	TBRC 7246	MF140755	MF140719	MF140810	MF140840
*A.tiankengensis*	KY11571	ON502848	ON502825	ON525446	ON525447
*A.tiankengensis*	KY11572	ON502821	ON502827	ON525448	ON525449
***A.tiankengensis***	**CQ05171**	**OQ300407**	**OQ300417**	**OQ349683**	**OQ325022**
***A.tiankengensis***	**CQ05172**	**OQ300408**	**OQ300419**	**OQ349690**	**OQ325023**
***A.tiankengensis***	**CQ05811**	**OQ300413**	**OQ300423**	**OQ349686**	**OQ325026**
***A.tiankengensis***	**CQ05812**	**OQ300414**	**OQ300425**	**OQ349687**	**OQ325027**
*A.tortricidarum*	BCC72638	MT356076	MT356088	MT477992	MT478004
*A.tortricidarum*	BCC41868	MT356077	MT356089	MT478008	MT477985
*A.tortricidarum*	BCC28583	MT356079	MT356090	MT477993	MT477986
*A.tuberculatus*	HUA186131	-	MF416521	-	MF416466
*A.waltergamsii*	TBRC7250	MF140749	MF140715	-	MF140835
*A.waltergamsii*	TBRC7251	MF140747	MF140713	MF140805	MF140833
*A.zaquensis*	HMAS 246917	MT789698	MT789696	-	MT797811
*A.zaquensis*	HMAS 246915	MT789699	MT789697	-	MT797812
*Samsoniellaaurantia*	TBRC7271	MF140764	MF140728	MF140818	MF140846
*S.aurantia*	TBRC7272	MF140763	MF140727	MF140817	MF140845

### ﻿Sequence alignment and phylogenetic analyses

DNASTAR Lasergene (version 6.0) was used to edit the DNA sequences. The ITS, LSU, RPB2 and TEF sequences were downloaded from GenBank, based on Sung et al. (2007), Kepler et al. (2017), Mongkolsamrit et al. (2018), Chen et al. (2018, 2019c, 2020b, 2020c, 2022a), Aini et al. (2020) and Wang et al. (2023) and others selected on the basis of BLAST searches in GenBank. ITS sequences and other loci were aligned and edited by MAFFT online service (Katoh et al. 2019) and MEGA6 (Tamura et al. 2013). Combined sequences of ITS, LSU, RPB2 and TEF were obtained using SequenceMatrix v.1.7.8 (Vaidya et al. 2011). The model was selected for Bayesian analysis by ModelFinder (Kalyaanamoorthy et al. 2017) in PhyloSuite software (Zhang et al. 2020).

The combined loci were analysed using Bayesian inference (BI) and maximum likelihood (ML) methods. For BI, a Markov chain Monte Carlo (MCMC) algorithm was used to generate phylogenetic trees with Bayesian probabilities using MrBayes v.3.2 (Ronquist et al. 2012) for the combined sequence datasets. The Bayesian analysis resulted in 20,001 trees after 10,000,000 generations. The first 4,000 trees, representing the burn-in phase of the analysis, were discarded, while the remaining 16,001 trees were used to calculate posterior probabilities in the majority rule consensus tree. After the analysis was finished, each run was examined using the programme Tracer v.1.5 (Drummond and Rambaut 2007) to determine burn-in and confirm that both runs had converged. ML analyses were performed with IQ-TREE (Trifinopoulos et al. 2016), using an automatic selection of the model. The final alignment and the original phylogenetic tree are available from TreeBASE under submission ID 30378.

### ﻿Genealogical Concordance Phylogenetic Species Recognition (GCPSR) analysis

The Genealogical Concordance Phylogenetic Species Recognition model was applied to analyse the related species. The pairwise homoplasy index (PHI) (Bruen et al. 2006) is a model test based on the fact that multiple gene phylogenies will be concordant between species and discordant due to recombination and mutations within a species. The test was performed in SplitsTree4 (Huson and Bryant 2006) as described by Quaedvlieg et al. (2014) to determine the recombination level within phylogenetically closely-related species using a four-locus concatenated dataset. The new species and their closely-related species were analysed using this model. The relationships between closely-related species were visualised by constructing a split graph, using both the LogDet transformation and splits decomposition options.

## ﻿Result

### ﻿Phylogenetic analyses

In the phylogenetic tree, *Samsoniellaaurantia* Mongkols., et al. (TBRC 7271 and TBRC 7272) was used as the outgroup. The concatenated sequences (ITS, LSU, RPB2 and TEF) included 23 species (49 strains) and consisted of 2,620 characters with gaps (ITS, 478; LSU, 745; RPB2, 717; and TEF, 680).

**Figure 1.**
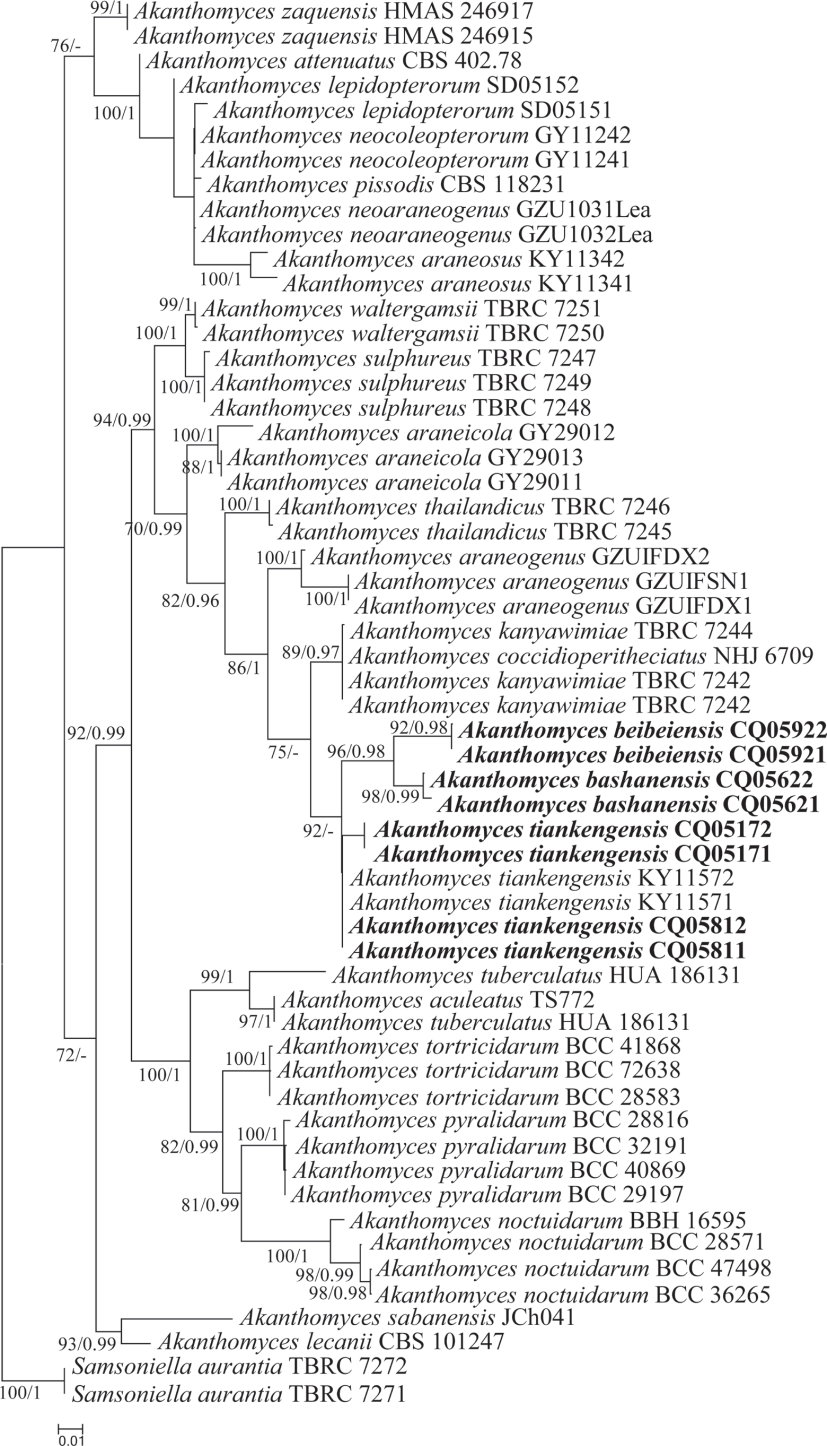
Phylogenetic relationships amongst the new strains and their allies based on multigene dataset (ITS, LSU, RPB2 and TEF). Statistical support values (≥70%/0.95) are shown at the nodes for ML bootstrap support/BI posterior probabilities.

The final value of the highest scoring tree was –11,790.345, which was obtained from the ML analysis of the dataset (ITS+LSU+RPB2+TEF). The parameters of the GTR model used to analyse the dataset were estimated, based on the following frequencies: A = 0.236, C = 0.283, G = 0.272, T = 0.209; substitution rates AC = 1.00000, AG = 2.12340, AT = 1.00000, CG = 1.00000, CT = 5.43884 and GT = 1.00000, as well as the gamma distribution shape parameter α = 0.557. The selected model for BI analysis was GTR+F+I+G4 (ITS+LSU+TEF) and K2P+G4 (RPB2). The phylogenetic trees (Fig. 1) constructed using ML and BI analyses were largely congruent and strongly supported in most branches. Phylogenetic analyses demonstrated that eight new strains formed a subclade with *Akanthomycestiankengensis* (KY11571 and KY11572) with high statistical support in ML analysis (92% ML). Strains CQ05171, CQ05172, CQ05811 and CQ05812 clustered with *A.tiankengensis* into a subclade, while the new species *A.beibeiensis* (CQ05921 and CQ05922) and *A.bashanensis* (CQ05621 and CQ05622) clustered in a subclade with high statistical support (96% ML/0.98 PP; Fig. 1).

### ﻿GCPSR analysis

**Figure 2.**
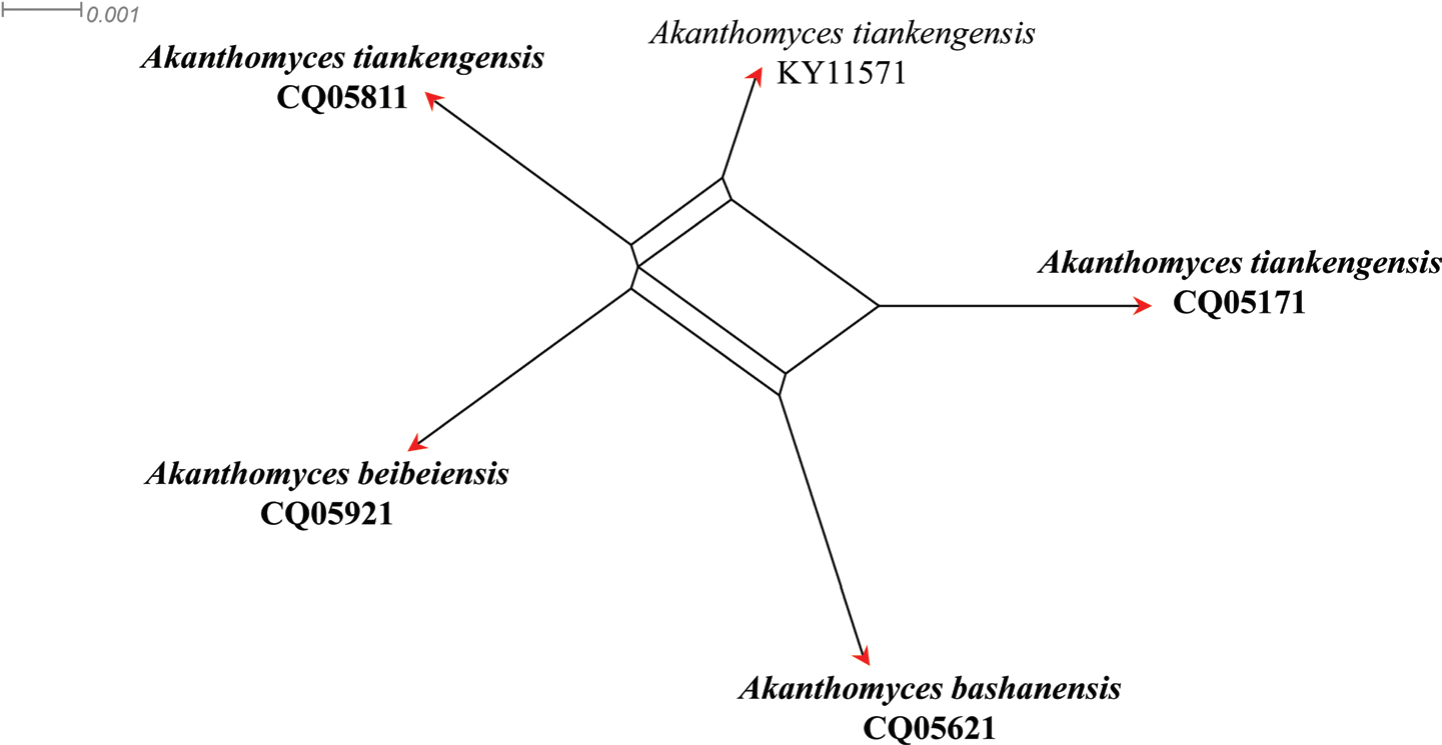
Results of the pairwise homoplasy index (PHI) test of closely-related species using both LogDet transformation and splits decomposition. PHI test results (Φ_w_) < 0.05 indicate significant recombination within the dataset. The new taxon is in bold type.

A four-locus concatenated dataset (ITS, LSU, RPB2 and TEF) was used to determine the recombination level within *Akanthomycesbashanensis* (CQ05621), *A.beibeiensis* (CQ05921) and *A.tiankengensis* (KY11571, CQ05171, CQ05811). Chaiwan et al. (2022) noted that, if the PHI is below the 0.05 threshold (Φw < 0.05), it indicates that there is significant recombination in the dataset, meaning that related species in a group and recombination level are not different. If the PHI is above the 0.05 threshold (Φw > 0.05), it indicates that it is not significant, which means the related species in a group level are different. The result of the pairwise homoplasy index (PHI) test of *A.bashanensis*, *A.beibeiensis* and *A.tiankengensis* was 0.333 and revealed that the three species were different (Fig. 2).

### ﻿Taxonomy

#### ﻿ Akanthomyces bashanensis

Taxon classificationFungiHypocrealesCordycipitaceae

W.H. Chen, Y.F. Han & J.D. Liang sp. nov.

07F0239E-420E-5EC7-A10F-54FD0F07399D

847339

Fig. 3

##### Type.

China, Chongqing, Beibei District, Jinyun Mountain (29°50'22.14959"N, 106°23'18.0744"E). On a dead spider (Araneae), 1 May 2021, Wanhao Chen, GZAC CQ0562 (holotype), ex-type living culture, CQ05621.

##### Description.

Spider host completely covered by white mycelium. Conidiophores mononematous, arising from the lateral hyphae. Colonies on PDA, attaining a diameter of 26–27 mm after 14 days at 25 °C, white, consisting of a basal felt, floccose hyphal overgrowth; reverse yellowish. Hyphae septate, hyaline, smooth-walled, 1.5–1.9 μm wide. Conidiophores mononematous, hyaline, smooth-walled, with single phialide or whorls of 2–4 phialides or verticillium-like from hyphae directly, 12.1–20.5 × 1.5–2.1 μm. Phialides consisting of a cylindrical, somewhat inflated base, 11.8–12.9 × 1.3–1.6 μm, tapering to a thin neck. Conidia hyaline, smooth-walled, fusiform to ellipsoidal, 1.7–2.6 × 1.6–1.8 μm, forming divergent and basipetal chains. Sexual state not observed.

**Figure 3.**
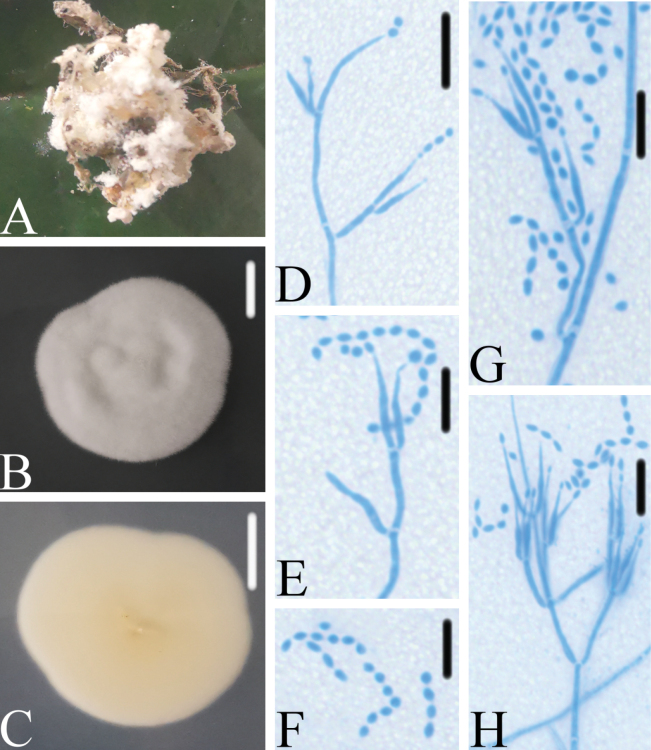
*Akanthomycesbashanensis***A** infected spider (Araneae) **B, C**PDA culture plate showing top (**B**) and reverse (**C**) sides of the colony **D–H** phialides and conidia. Scale bars: 10 mm (**B, C**); 10 μm (**D–H**).

##### Etymology.

Referring to its location in Jinyun Mountain, which was formerly known as Bashan.

##### Additional strain examined.

China, Chongqing, Beibei District, Jinyun Mountain (29°50'22.14959"N, 106°23'18.0744"E). On a dead spider (Araneae), 1 May 2021, Wanhao Chen, CQ05622.

##### Remarks.

*Akanthomycesbashanensis* was easily identified as *Akanthomyces*, based on the BLASTn result in NCBI and the phylogenetic analysis of combined datasets (ITS, LSU, RPB2, TEF) (Fig. 1) and it has a close relationship with another new species, *A.beibeiensis*. *A.bashanensis* was easily distinguished from *A.beibeiensis* by its longer phialides and smaller conidia. Jeewon and Hyde (2016) recommended that a minimum of > 1.5% nucleotide differences in the ITS regions and protein coding genes may be indicative of a new species. The pairwise dissimilarities of ITS, LSU, RPB2 and TEF sequences show 11 bp differences within 569 bp (1.93%), 19 bp differences within 881 bp (2.15%), 13 bp differences within 1070 bp (1.21%) and 4 bp differences within 973 bp (0.41%) between *A.bashanensis* and *A.beibeiensis*, respectively. The pairwise dissimilarities of ITS, LSU, RPB2 and TEF sequences show 10 bp differences within 569 bp (1.75%), 20 bp differences within 881 bp (2.27%), 19 bp differences within 1070 bp (1.77%) and 4 bp differences within 973 bp (0.41%) between *A.bashanensis* and *A.tiankengensis*, respectively. Furthermore, *A.aranearum* (Petch) Mains and *A.ryukyuensis* (Kobayasi & Shimizu) Mongkols., Noisrip., Thanakitp., Spatafora & Luangsa-ard were both absent from the available sequence in NCBI and having a spider host. Comparing with the typical characteristics (Table 2), *A.bashanensis* was easily distinguished from *A.aranearum* by its cylindrical phialide, smaller fusiform to ellipsoidal conidia and absence of synnemata and distinguished from *A.ryukyuensis* by absence of teleomorphs. Thus, the morphological characteristics and molecular phylogenetic results support *A.bashanensis* as a new species.

**Table 2.** Morphological comparison of two new species with other related *Akanthomyces* species.

Species	Synnemata (mm)/ Perithecia (μm)	Conidiophores (μm)	Phialides (μm)/ Asci (μm)	Conidia (μm)/ Part-spores (μm)	Reference
*Akanthomycesaranearum*	Cylindrical to clavate, 0.8–10 × 0.1–0.2, brown		Obovoid or ellipsoid 6–12 × 4–8	Narrowly obclavate, 8–14 × 1.5–3	Mains (1950)
*Akanthomycesbashanensis*	Synnemata not observed	Mononematous, 12.1–20.5 × 1.5–2.1.	cylindrical, somewhat inflated base, 11.8–12.9 × 1.3–1.6	fusiform to ellipsoidal, 1.7–2.6 × 1.6–1.8	This study
*Akanthomycesbeibeiensis*	Synnemata not observed	Mononematous, 14.2–19.4 × 1.0–2.1	cylindrical, somewhat inflated base, 7.0–9.2 × 2.1–2.5	fusiform to ellipsoidal, 2.0–3.3 × 2.0–2.6	This study
*Akanthomycesryukyuensis*	Synnemata not observed/ Pyriformia, 570–630 × 170–250	Conidiophores not observed	Phialides not observed/5 wide, cap 3 wide	Conidia not observed/ 1 × 1–4	Kobayasi and Shimizu (1982)
*Akanthomycestiankengensis*	Synnemata not observed	Erect, usually arising from the aerial hyphae	13.9–17.1 × 1.1–1.6 with a cylindrical basal portion	Fusiform, 2.3–3.0 × 1.5–2.3	Chen et al. (2022a)

#### ﻿ Akanthomyces beibeiensis

Taxon classificationFungiHypocrealesCordycipitaceae

W.H. Chen, Y.F. Han & J.D. Liang sp. nov.

5C544799-C9EC-5E4C-AF0E-CBA47AA982FF

847340

Fig. 4

##### Type.

China, Chongqing, Beibei District, Jinyun Mountain (29°50'22.14959"N, 106°23'18.0744"E). On a dead spider (Araneae), 1 May 2021, Wanhao Chen, GZAC CQ0592 (holotype), ex-type living cultures, CQ05921.

##### Description.

Spider host completely covered by white mycelium. Colonies on PDA, attaining a diameter of 34–37 mm after 14 days at 25 °C, white, consisting of a basal felt, floccose hyphal overgrowth; reverse yellowish. Hyphae septate, hyaline, smooth-walled, 1.4–1.9 μm wide. Conidiophores mononematous, hyaline, smooth-walled, with single phialide or whorls of 2–6 phialides or verticillium-like from hyphae directly, 14.2–19.4 × 1.0–2.1 μm. Phialides consisting of a cylindrical, somewhat inflated base, 7.0–9.2 × 2.1–2.5 μm, tapering to a thin neck. Conidia hyaline, smooth-walled, fusiform to ellipsoidal, 2.0–3.3 × 2.0–2.6 μm, forming divergent and basipetal chains. Sexual state not observed.

**Figure 4.**
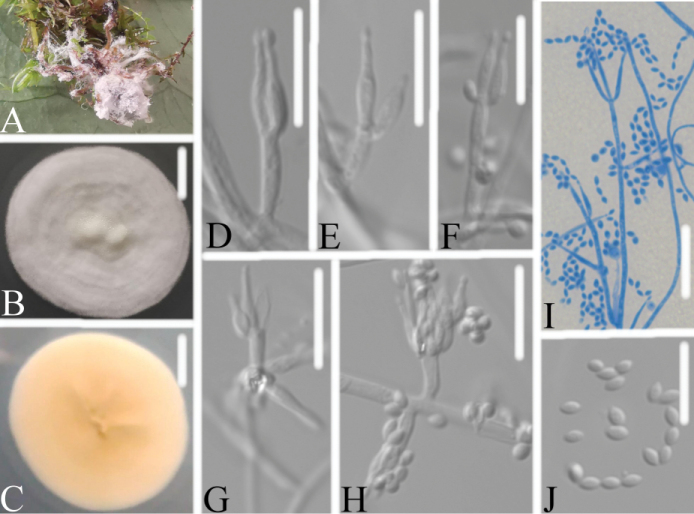
*Akanthomycesbeibeiensis***A** infected spider (Araneae) **B, C**PDA culture plate showing top (**B**) and reverse (**C**) sides of the colony **D–J** phialides and conidia. Scale bars: 10 mm (**B, C**); 10 μm (**D–J**).

##### Etymology.

Referring to its location in Beibei District.

##### Additional strain examined.

China, Chongqing, Beibei District, Jinyun Mountain (29°50'22.14959"N, 106°23'18.0744"E). On a dead spider (Araneae), 1 May 2021, Wanhao Chen, CQ05692.

##### Remarks.

*Akanthomycesbeibeiensis* was easily identified as *Akanthomyces* according to the blast result in NCBI and the phylogenetic analysis of combined datasets (ITS, LSU, RPB2, TEF) (Fig. 1) and it has a close relationship with another new species, *A.bashanensis*. *A.beibeiensis* is easily distinguished from *A.bashanensis* by its shorter phialide and larger conidia. The pairwise dissimilarities of ITS, LSU, RPB2 and TEF sequences show 4 bp differences within 569 bp (0.7%), 8 bp differences within 881 bp (0.9%), 20 bp differences within 1070 bp (1.86%) and 2 bp differences within 973 bp (0.2%) between *A.beibeiensis* and *A.tiankengensis*, respectively. Furthermore, *A.aranearum* and *A.ryukyuensis* were both absent from the available sequences in NCBI and had spider hosts. Comparing with the typical characteristics (Table 2), *A.beibeiensis* was easily distinguished from *A.aranearum* by its cylindrical phialide, smaller fusiform to ellipsoidal conidia and absence of synnemata, and distinguished from *A.ryukyuensis* by absence of teleomorphs. Thus, the morphological characteristics and molecular phylogenetic results support *A.beibeiensis* as a new species.

#### ﻿ Akanthomyces tiankengensis

Taxon classificationFungiHypocrealesCordycipitaceae

W.H. Chen, Y.F. Han, J.D. Liang & Z.Q. Liang, Microbiology Spectrum 10(5): e01975-22

D507F260-E51B-5C2F-97F1-1338749100CF

Fig. 5

##### Description.

Spider host completely covered by white mycelium. Colonies on PDA, attaining a diameter of 27–28 mm after 14 days at 25 °C, white, consisting of a basal felt, floccose hyphal overgrowth; reverse yellowish. Hyphae septate, hyaline, smooth-walled, 2.4–2.6 μm wide. Conidiophores mononematous, hyaline, smooth-walled, with single phialide or whorls of 2 phialides. Phialides consisting of a cylindrical, somewhat inflated base, 16.2–25.3 × 2.1–2.9 μm, tapering to a thin neck. Conidia hyaline, smooth-walled, subglobose to ellipsoidal, 2.4–3.8 × 2.1–3.0 μm, forming divergent and basipetal chains. Sexual state not observed.

**Figure 5.**
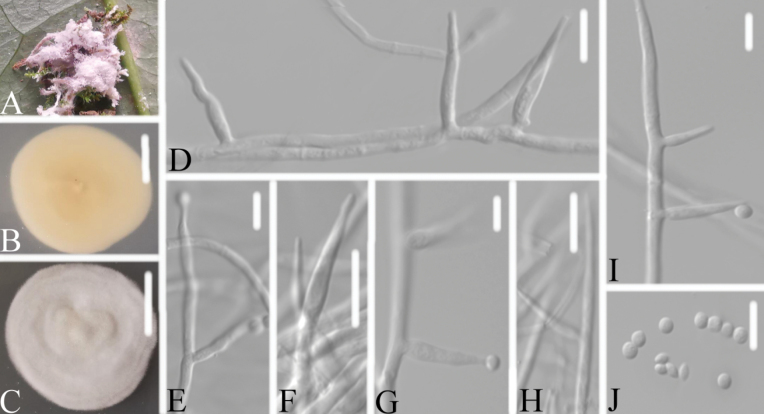
*Akanthomycestiankengensis***A** infected spider (Araneae) **B, C**PDA culture plate showing reverse (**B**) and top (**C**) sides of the colony **D–J** phialides and conidia. Scale bars: 10 mm (**B, C**); 10 μm (**D–J**).

##### Strains and specimen examined.

China, Chongqing, Beibei District, Jinyun Mountain (29°50'22.14959"N, 106°23'18.0744"E). On a dead spider (Araneae), 1 May 2021, Wanhao Chen, GZAC CQ0581, living cultures, CQ05811 and CQ05812; GZAC CQ0517, living cultures, CQ05171 and CQ05172; GZAC CQ0501, CQ0509, CQ0510, CQ0512, CQ0516, CQ0558, CQ0582, CQ0586, CQ0598, CQ0590.

##### Remarks.

Strains CQ05811 and CQ05812 were identified as belonging to *Akanthomyces*, based on the phylogenetic analyses (Fig. 1) and clustered with *A.tiankengensis* in a subclade. The characteristics of CQ05811 and CQ05812 were very closely linked with *A.tiankengensis*, which had fusiform conidia (2.3–3.0 × 1.5–2.3 μm) and shorter phialide (13.9–17.1 × 1.1–1.6 μm). Furthermore, the pairwise dissimilarities of ITS sequences show 3 bp difference within 569 bp between CQ05811 and *A.tiankengensis* (0.52%) and no difference within 569 bp between CQ05171, CQ05172 and *A.tiankengensis*. Thus, molecular phylogenetic results and morphologically based conclusions supported the idea that strains CQ05811, CQ05812, CQ05171 and CQ05172 were *A.tiankengensis*.

## ﻿Discussion

*Akanthomyces* species are widely distributed and commonly isolated from soil, insects and spiders (Chen et al. 2018, 2019c; Shrestha et al. 2019). Amongst the 33 species, *A.aranearum* (Petch) Mains, *A.araneicola* W.H. Chen, et al., *A.araneogenus* Z.Q. Liang, W.H. Chen & Y.F. Han, *A.araneosus* W.H. Chen, Y.F. Han, J.D. Liang & Z.Q. Liang, *A.coccidioperitheciatus* (Kobayasi & Shimizu) Spatafora, Kepler & B. Shrestha, *A.kanyawimiae* Mongkols., Noisrip., Thanakitp., Spatafora & Luangsa-ard, *A.neoaraneogenus* (W.H. Chen et al.) W.H. Chen et al., *A.lecanii* (Zimm.) Spatafora, Kepler & B. Shrestha, *A.ryukyuensis* (Kobayasi & Shimizu) Mongkols., Noisrip., Thanakitp., Spatafora & Luangsa-ard, *A.sulphureus* Mongkols., Noisrip., Thanakitp., Spatafora & Luangsa-ard, *A.thailandicus* Mongkols., Spatafora & Luangsa-ard, *A.tiankengensis* W.H. Chen, et al. and *A.waltergamsii* Mongkols., Noisrip., Thanakitp., Spatafora & Luangsa-ard have been reported to be spider-associated fungi.

In the present study, the new strains differed from other spider-pathogenic species and had a close relationship with *Akanthomycestiankengensis*, based on the phylogenetic analysis. Two new species were established by combining phylogenetic analysis and morphological characteristics. Interestingly, *A.tiankengensis* was located at Monkey-Ear Tiankeng and found in November, indicating that it had adapted to the cold environment. Whether these new species can adapt to their environment and have special metabolic processes is worthy of further research.

The hosts of *Akanthomyces* species cover Hemiptera, Coleoptera, Lepidoptera, Orthoptera and Araneae (Hodge 2003; Mongkolsamrit et al. 2018; Chen et al. 2020b, 2022c). Chen et al. (2022b) noted that a host jump may be common in *Simplicillium* species, the spider-associated species may have originated from insects and then jumped to a spider host. An abundant diversity in insects and spiders has been discovered at Jinyun Mountain (Huang and Zhang 1991; Li et al. 2009a, 2009b; Wang et al. 2012; Huang et al. 2021; Yan et al. 2021). Whether the new species originally came from an insect host or other substrates and then jumped to a spider host, is also worthy of further research.

Mains (1950) and Vincent et al. (1988) surmised that cylindrical synnemata covered by a hymenium-like layer of phialides producing one-celled catenulate conidia were the typical characteristics of *Akanthomyces*. However, Chen et al. (2020b, 2020c) reported two new *Akanthomyces* species with mononematous conidiophores. In the present study, the two new species had mononematous conidiophores. *Akanthomyces* species with mononematous conidiophores are often present on the surface of moss or in open places, from which their conidia can easily be spread by airflow diffusion or other methods. Those *Akanthomyces* species with synnematous conidiophores often appear in the shrubbery of the original forest, litter layer or shallow soil (Hywel-Jones 1996), where air flow under the forest canopy is slow and humidity is high and where dispersal of conidia through airflow diffusion is difficult. Therefore, the presence of synnematous conidiophores may be the result of convergent evolution, which could help them to fit in their niche (Abbott 2002). Thus, the *Akanthomyces* species may change their type of conidiophores to increase their adaptability to different environmental conditions.

The taxonomic delimitation of *Akanthomyces* was originally based on morphological characteristics. Kepler et al. (2017) proposed the rejection of *Torrubiella* Boud. and *Lecanicillium* W. Gams & Zare in favour of *Akanthomyces* and transferred *Torrubiella* and *Lecanicillium* species into *Akanthomyces*, which has resulted in a combined analysis of morphological characteristics and phylogenetic analysis for the taxonomy of *Akanthomyces*. In this research, a PHI test and base difference rate were added, which could solve the taxonomic delimitation of cryptic species. Amongst the four loci (ITS, LSU, RPB2 and TEF), the locus TEF could not be used to distinguish *A.bashanensis*, *A.beibeiensis* and *A.tiankengensis*. However, any two of the three loci could easily be used to distinguish these three species. Thus, we recommend that at least two loci should be provided for the cryptic *Akanthomyces* species and analysis of the cryptic species with its related species should be done using the multiple methods. Furthermore, the genomics data, phylogenetic networks and haplotype analysis should be applied to cryptic species and the taxonomy of *Akanthomyces* made it closer to the natural taxonomy system.

Currently, the diversity of entomopathogenic fungi in some Natural Reserves and Forest Parks in different regions of China has shown that the abundant diversity of entomopathogenic fungi is present in the study areas, and there is a high species diversity in specific areas (Chen et al. 2019a, 2020a; Fan et al. 2020; Zhao et al. 2020, 2021; Zhang et al. 2021). Jiyun Mountain is located in Chongqing City and has varied altitudes, abundant plant and animal resources, which have nurtured abundant fungal resources (Zhou et al. 2012a, b). In this research, abundant *Akanthomyces* specimens were found at Jinyun Mountain and further attention needs to be paid to the diversity of other entomopathogenic fungi in Chongqing, China.

## Supplementary Material

XML Treatment for Akanthomyces bashanensis

XML Treatment for Akanthomyces beibeiensis

XML Treatment for Akanthomyces tiankengensis

## ﻿Additional information

### Author ORCIDs

Wan-Hao Chen https://orcid.org/0000-0001-7240-6841

Jian-Dong Liang https://orcid.org/0000-0002-3939-3900

Yan-Feng Han https://orcid.org/0000-0002-8646-3975

### Data availability

All of the data that support the findings of this study are available in the main text.
